# Study of far-field reduction in high power 940 nm vertical-cavity surface-emitting lasers cascaded by tunnel junctions

**DOI:** 10.1038/s41598-025-17744-1

**Published:** 2025-09-01

**Authors:** Wei-Hao Huang, Chi-Yao Shan, Kai-Lun Chi, Yi-Jun Xu, Tien-Chang Lu

**Affiliations:** 1 Department of Photonics, College of Electrical and Computer Engineering, National Yang Ming Chiao Tung University, Hsinchu, 30010 Taiwan; 2 Department of Opto-Electronic Development Center, WIN Semiconductor Corporation, Taoyuan, Taiwan

**Keywords:** Optics and photonics, Lasers, LEDs and light sources, Diode lasers, Semiconductor lasers, Solid-state lasers

## Abstract

This paper characterizes the performance of 940 nm single-junction (1 J) and triple-junction (3 J) vertical-cavity surface-emitting laser (VCSEL) arrays, tested at room temperature under 1.8 ns pulsed current injection. By suppressing thermal effects, the slope efficiency (SE) of the 1 J VCSEL array reaches 1.05 W/A, while the 3 J VCSEL array achieves 3.2 W/A, with a peak output power exceeding 120 W, demonstrating a significant performance enhancement. Furthermore, we observe that in the 3 J VCSEL array, the far-field (FF) divergence angle gradually decreases with increasing injection current, reducing from approximately 17° to about 5°. The far-field beam profile exhibits a Gaussian distribution, and spectral measurements indicate that the fundamental mode is dominant. We further analyze the characteristics of the VCSEL through both simulations and measurements. Current path analysis reveals that in the 3 J structure, the presence of a highly doped tunnel junction (TJ) and multiple oxide layers alleviates current crowding compared to the 1 J structure, resulting in a different gain distribution. Calculations show that the overlap between the gain region and the fundamental mode is greater than that of higher-order modes, which may explain the dominance of the fundamental mode. The results from single-device testing align with the observations in the VCSEL array, consistently demonstrating fundamental mode dominance. This phenomenon contributes to a reduced divergence angle, presenting a significant advantage for future optoelectronic applications.

## Introduction

In recent years, VCSELs have become a focal point of development in both academia and industry, with widespread applications in 3D sensing^1–3^ optical communication^4–6^ consumer electronics^7–10^ and industrial processing^11,12^. The key advantages of VCSELs include their circular beam profile, low divergence angle, high efficiency, high modulation speed^13,14^ and compatibility with large-scale two-dimensional array fabrication^15–17^ making them highly competitive for high-power sensing applications such as LiDAR.

With advancements in semiconductor growth and fabrication technologies, the performance and application scope of VCSELs continue to expand. By integrating a tunnel junction (TJ)—a highly doped (> 10¹⁹ cm⁻³) and ultrathin PN junction—active regions can be vertically stacked, enabling carrier recycling between adjacent active regions^18,19^. This approach has been demonstrated to effectively enhance output power and differential quantum efficiency^20–22^. Additionally, increasing the gain volume in multi-junction(MJ) VCSELs improves the round-trip gain of the optical field^23,24^ thereby reducing the requirement of high reflectivity of the output mirror while maintaining the laser threshold current. In recent years, Zhan et al. demonstrated a 7-junction VCSEL incorporating an anti-reflective cavity design, which delivered a small beam divergence of 8 degree^25^. Work by Li et al. primarily discusses the thermal distribution and the performance difference between multi-junction and conventional VCSELs^26^. Another study focuses on the impact of TJ lasers with high-Al-content barrier layers on the modal behavior^27^. Additionally, a study of theoretical model showing how variations in TJ doping concentration alter the spatial carrier distribution^28^. However, in multi-junction structures, the increased number of oxide layers enhances the refractive index contrast between oxidized and non-oxidized regions, leading to an increased number of optical modes and a wider divergence angle, which negatively impacts sensing applications^29,30^.

In this study, we investigate the electrical and optical characteristics of 1 J and triple-junction 3 J VCSEL structures. In the 3 J VCSEL array, we achieve a peak output power exceeding 120 W using short-pulse current injection. Intriguingly, we observe that as the driving current increases, the divergence angle gradually decreases to 11°. Spectral analysis indicates that this phenomenon is primarily attributed to the dominance of the fundamental mode. To further understand this behavior, we develop a theoretical model and simulate the current paths of both structures under different injection conditions, analyzing the distribution of gain and optical modes. The mitigation of current crowding by applying highly doped tunnel junctions could be the main factor. Finally, we measure the spectral and far-field characteristics of individual VCSEL devices under continuous-wave (CW) operation. The results are consistent with our theoretical model, successfully explaining the near-field, far-field, and output characteristics of the 3 J VCSEL. Moreover, the observations from individual VCSEL devices align with those from the array, further validating the mechanism governing array performance.

## Experiment

**Fig. 1:**
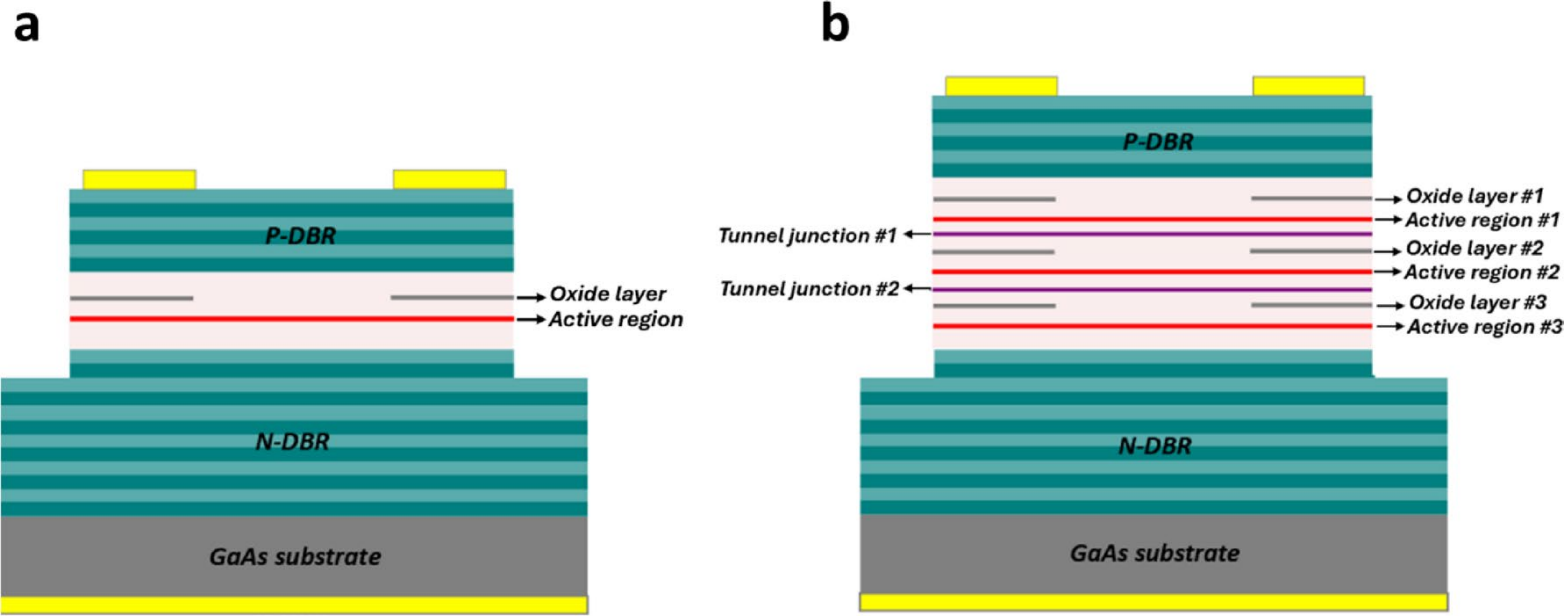
Cross-section illustration of (**a**) single-junction and (**b**) three-junctions VCSEL.

The schematic diagrams of the 1 J and 3 J VCSELs are shown in Fig. 1a and b, respectively. The complete 1 J structure consists of a p-type distributed Bragg reflector (DBR), an active region, and an n-type DBR. Each DBR pair is composed of Al_0.9_Ga_0.1_As/Al_0.1_Ga_0.9_As layers, each with a quarter-wavelength thickness, providing a refractive index contrast that forms high-reflectivity mirrors. Since the number of p-type DBR layers is lower than that of the n-type DBR, the laser emission is directed upwards. In this design, an oxide layer is placed above the active region to provide lateral electrical and optical confinement.

The active region consists of three pairs of compressively strained InGaAs/GaAs quantum wells designed to achieve high optical gain at 940 nm. Additionally, the active region is positioned at the electric field peak to enhance optical gain, while the oxide layer is placed at the node to minimize internal losses^31,32^. As shown in Fig. 1b, the 3 J structure is similar to the 1 J design but consists of multiple oxide layers and active regions connected via tunnel junctions. It is worth noting that, due to the highly doped nature of tunnel junctions, they must be carefully placed at the electric field node to avoid excessive free carrier absorption losses.

First, the top p-type ohmic contact metal is deposited using electron beam evaporation. Subsequently, inductively coupled plasma reactive-ion etching (ICP-RIE) is used for dry etching to define the mesa structure. A wet oxidation process is then performed to form a 10 μm oxidation aperture. After the planarization process, an interconnect metal layer is deposited on the p-type contact metal to serve as a probing pad. Finally, backside metallization is performed to form the n-type ohmic contact, completing the device fabrication.

**Fig. 2:**
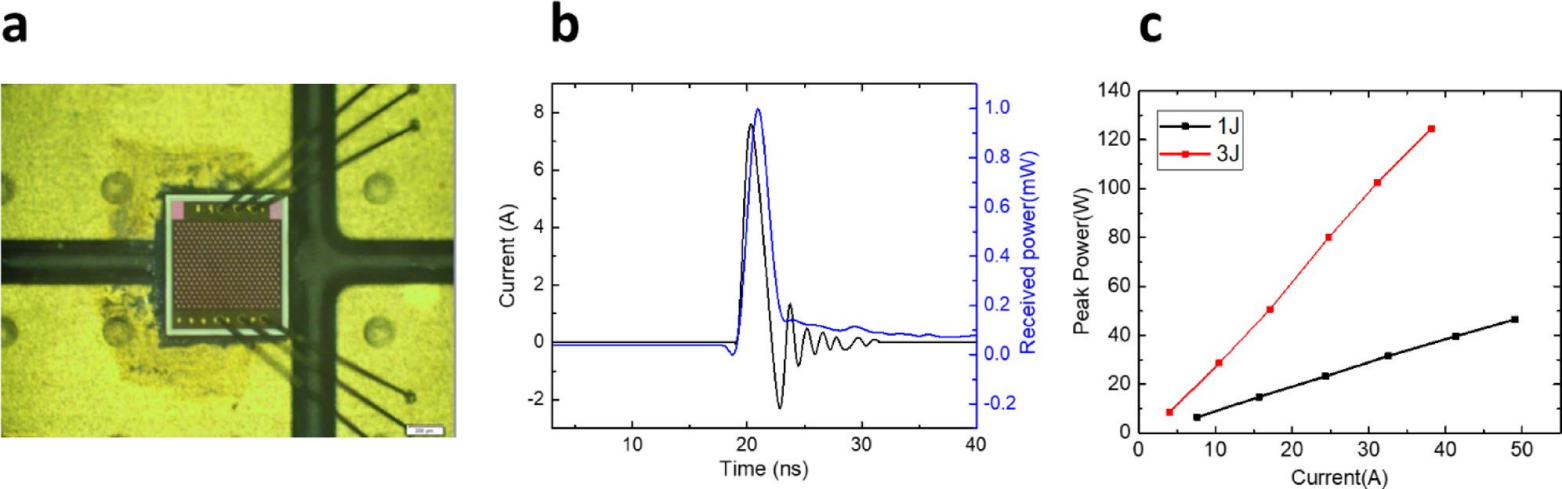
(**a**) Optical microscope top view image of a 380 elements VCSEL array on driver board, the length of scale bar is 400 μm (**b**) The 7.45 A current signal (1.8 ns pulse width, 0.05% duty cycle) pulsed into the VCSEL array and the corresponding output light received by PD (**c**) the measured L-I curve of both structures.

In our previous study^33^ we demonstrated a 380-element VCSEL array utilizing a chip-on-board (COB) packaging approach to achieve short-pulse injection, as shown in Fig. 2a. To ensure the accuracy of the input electrical and output optical waveforms, a high-speed photodiode (UPD-35-UVIR-P) and an oscilloscope were used for measurements. As illustrated in Fig. 2b, both the input and output waveforms exhibit a full width at half maximum (FWHM) of approximately 1.8 nanoseconds, with minimal rise and fall times, confirming proper impedance matching of the RLC circuit under COB packaging conditions.

The light-current (L-I) characteristics of the VCSEL arrays were measured at 25 °C, with the results shown in Fig. 2c. Optical power measurements were performed using a time-averaging photodiode (PD10-C), where the average power was first measured, and the peak power was subsequently calculated by dividing by the pulse duty cycle. For the 1 J structure, a slope efficiency (SE) of approximately 1.05 W/A was measured, reaching a peak power of 50 W at an injection current of 50 A. In contrast, the 3 J structure exhibited an SE of 3.2 W/A, achieving a maximum peak power of approximately 120 W at 38 A. These results clearly demonstrate the scaling of power output with an increasing number of junctions.

**Fig. 3:**
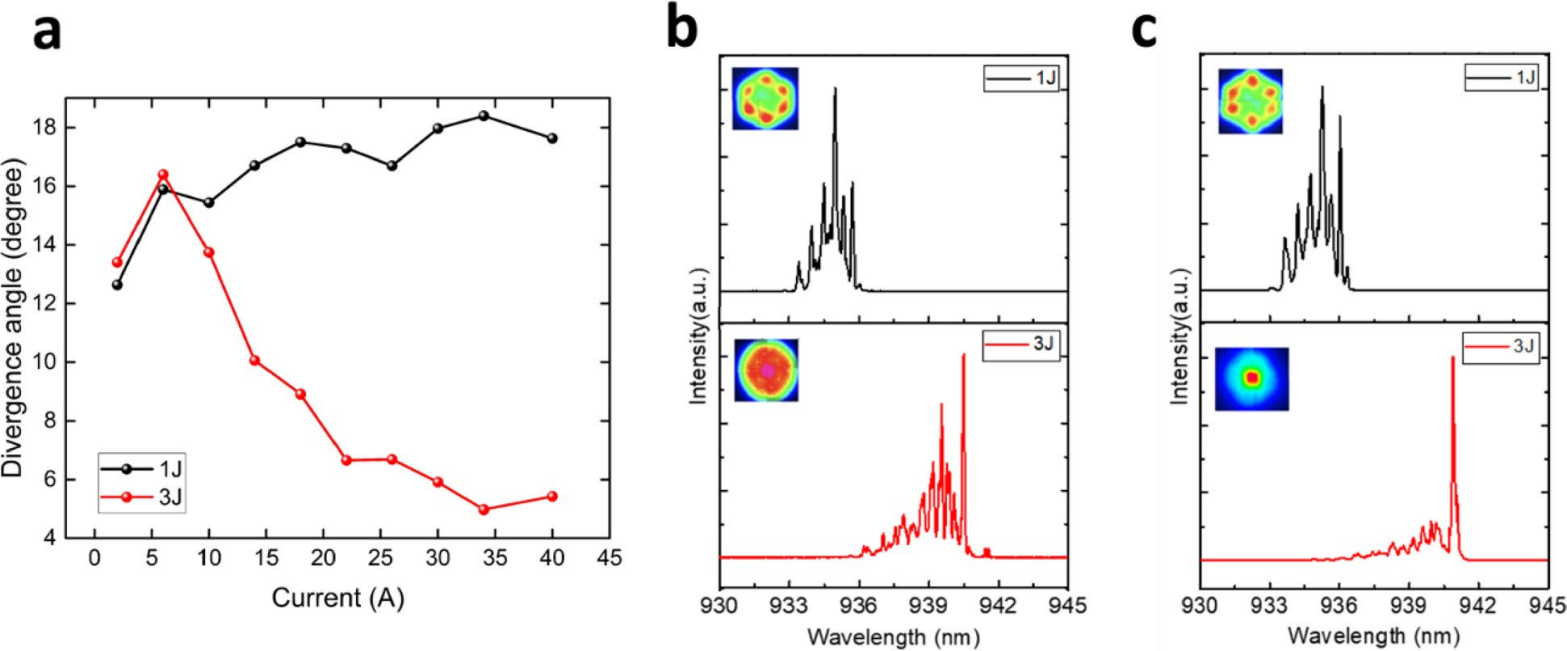
(**a**) Far-field as a function of injection current for the 1 J and 3 J VCSEL arrays. (**b**) and (**c**) show the corresponding emission spectra measured at 10 A and 40 A, respectively, for the same 1 J and 3 J arrays. The insets in (**b**) and (**c**) present the associated two-dimensional far-field beam profiles.

The variation in far-field divergence angles for different bias currents in both structures is shown in Fig. 3a. In the 1 J structure, the divergence angle gradually increases with increasing injection current. However, an interesting phenomenon is observed in the 3 J structure: the divergence angle initially increases with current up to 7 A but subsequently decreases as the current continues to rise, reaching a minimum of 5 degrees at 34 A.

To further investigate the underlying cause of this phenomenon, we measured the emission spectra under different injection currents, as shown in Fig. 3b and c. In the 1 J structure, higher-order modes are more dominant than the fundamental mode. The high intensity in the shorter wavelength region results in side lobes in the far-field distribution, a commonly observed phenomenon in conventional single-junction VCSELs operating at high currents, which is generally considered undesirable for beam quality. In contrast, the suppression of higher-order modes is more pronounced in the 3 J array. At an operating current of 40 A, the spectrum becomes significantly cleaner, with the fundamental mode at the longest wavelength dominating. This result strongly suggests that the gradual reduction in divergence angle is primarily due to the suppression of higher order modes. Since the presence of an array increases the complexity of spectral, near-field, and far-field analysis, the next section will conduct simulations and experiments on individual 1 J and 3 J VCSEL devices to gain a deeper understanding of this phenomenon.

## Results and discussion

**Fig. 4:**
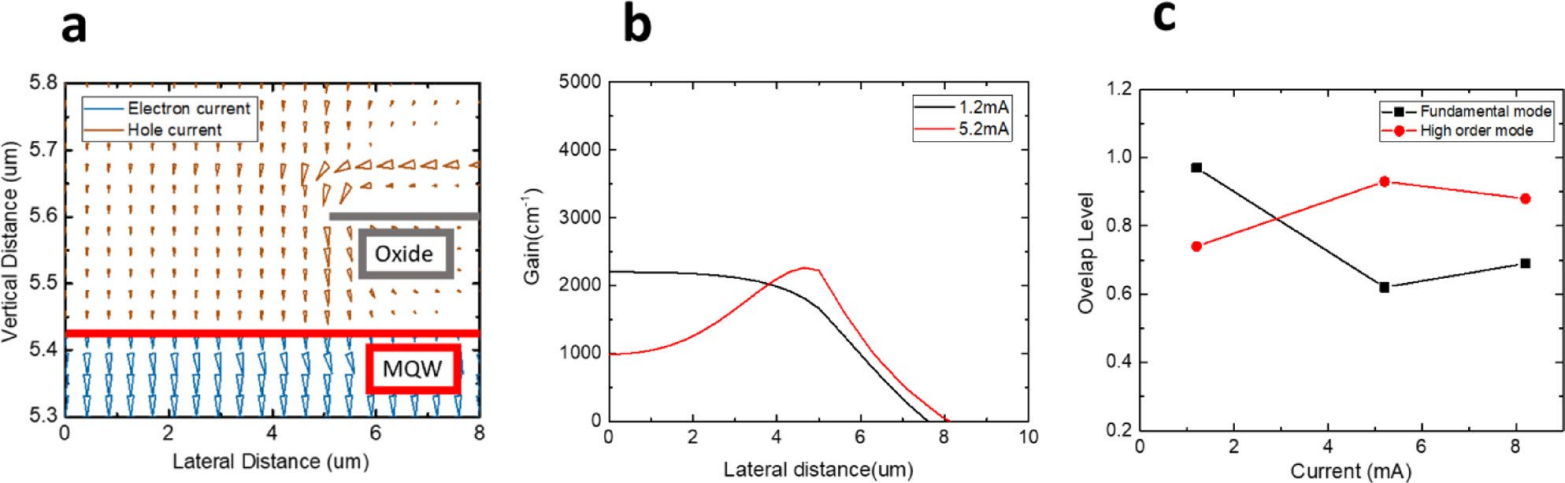
(**a**) 1 J structure simulated current path under 1.2 mA. (**b**) The 1-D gain profile at different bias and (**c**) the overlap level of gain profile between fundamental mode and high order mode.

To examine the differences in current transport paths and gain distributions between 1 J and 3 J VCSELs, this study utilizes the PICS3D software to simulate both configurations. The structural model is constructed based on the descriptions provided in the previous section. To streamline the analysis, this discussion focuses on the characteristics of a single device. Figure 4a presents the two-dimensional current distribution in the 1 J structure. In the illustration, the oxidized region appears as a gray layer with an aperture radius of 5 μm, positioned above the active region. Since the aperture diameter is smaller than the inner diameter of the electrode, a higher current density emerges at the aperture edges, a phenomenon known as current crowding^34^. This effect becomes more pronounced as the injection current increases. Additionally, spatial hole burning^35^ further influences the current distribution, ultimately leading to a higher current density near the oxidized aperture (OA). The lateral variation of the gain distribution in the active region is depicted in Fig. 4b. At a bias current of 1.2 mA, the gain is initially uniform within the aperture but gradually decreases beyond it. However, as the bias current increases, the interplay of the aforementioned effects results in a reduction in gain at the center relative to the edges. To explore the correlation between gain distribution and optical modes, the overlap integral between these two factors was computed, quantifying how effectively each mode utilizes the available gain, as illustrated in Fig. 4c. The overlap level is calculated by.

1 $${\text{Overlap}}\;\,{\text{level = }}\frac{{\int {f(x) \cdot g(x)\;dx} }}{{\sqrt {\int {f(x)^{2} \;dx} } \cdot \sqrt {\int {g(x)^{2} dx} } }}$$

where *f*(*x*) and *g*(*x*) are the mode and gain distribution function along radius *x* direction. According to the theory of linear polarization, the fundamental mode exhibits a Gaussian intensity profile, primarily concentrated at the waveguide’s center, whereas higher-order modes display intensity peaks near the edges. Simulation results reveal that at low current levels, the fundamental mode remains dominant. However, as the bias current reaches approximately 3 mA, the mode overlaps characteristics shift, indicating a transition in dominance toward higher-order modes beyond this threshold.

**Fig. 5:**
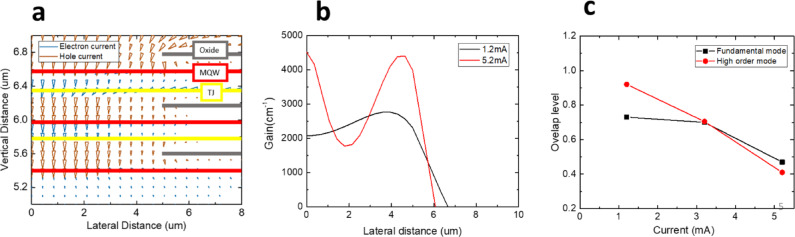
(**a**) 3 J structure simulated current path under 1.2 mA. (**b**) The 1-D gain profile at different bias and (**c**) the overlap level of gain profile between fundamental mode and high order mode.

A detailed analysis of the 3 J structure is presented in Fig. 5. As shown in Fig. 5a, the current crowding effect remains evident around the first active layer. However, in the two lower active layers, the current is predominantly confined within the oxidized region and propagates vertically downward, effectively mitigating the current crowding effect. This improvement can be attributed to two primary factors. First, the initial oxidation aperture restricts most of the injected current from the outer electrode within the oxidized region, leading to a more concentrated current distribution. Second, after traversing the highly doped tunnel junction layer, the carrier distribution becomes more uniform, likely due to the presence of highly doped electrons within the tunnel junction. This high doping level reduces resistance and enhances lateral current spreading, as clearly observed in the two lower active layers. Figure 5b illustrates the lateral gain distribution across the third active layers under varying bias currents. At 1.2 mA, the gain is initially uniform within the aperture. However, as the bias current increases, the gain profile exhibits a double-peaked distribution, with maxima at both the center and the edges of the oxide layer. This phenomenon arises from current crowding in the first oxide layer, which amplifies gain near the edges, while a portion of the confined current redistributes into the lower two active layers, resulting in peak gain at the center. Following the methodology applied in the 1 J structure, the overlap integral between the gain distribution and optical modes was computed to quantify the extent to which each mode benefits from the available gain, as depicted in Fig. 5c. The results reveal that in the 3 J structure, higher-order modes dominate at low current levels. However, beyond 3 mA, a transition occurs, with the fundamental mode becoming dominant. This modal shift subsequently influences the emission divergence angle.

**Fig. 6:**
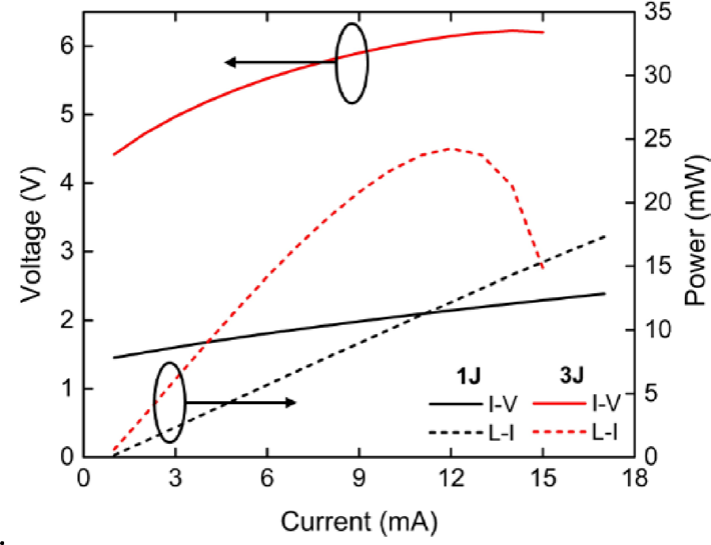
The measured L-I-V characteristics of the 1 J and 3 J structures.

To validate the simulation results, experimental measurements and analyses were conducted on a single device under CW operation. Figure 6 presents the L-I-V characteristics of a single emitter for both the 1 J and 3 J structures. For the 1 J structure, SE of approximately 1.11 W/A was measured, along with threshold current of 0.92 mA, threshold voltage of approximately 1.45 V, and the series resistance of approximately 60 Ω. No thermal roll-over was observed up to an injection current of 17 mA. In contrast, the 3 J structure exhibited higher SE of 2.73 W/A, lower threshold current of 0.76 mA, threshold voltage of 4.42 V, and a series resistance of approximately 132 Ω. Thermal roll-over occurred at 11 mA due to increased self-heating.

**Fig. 7:**
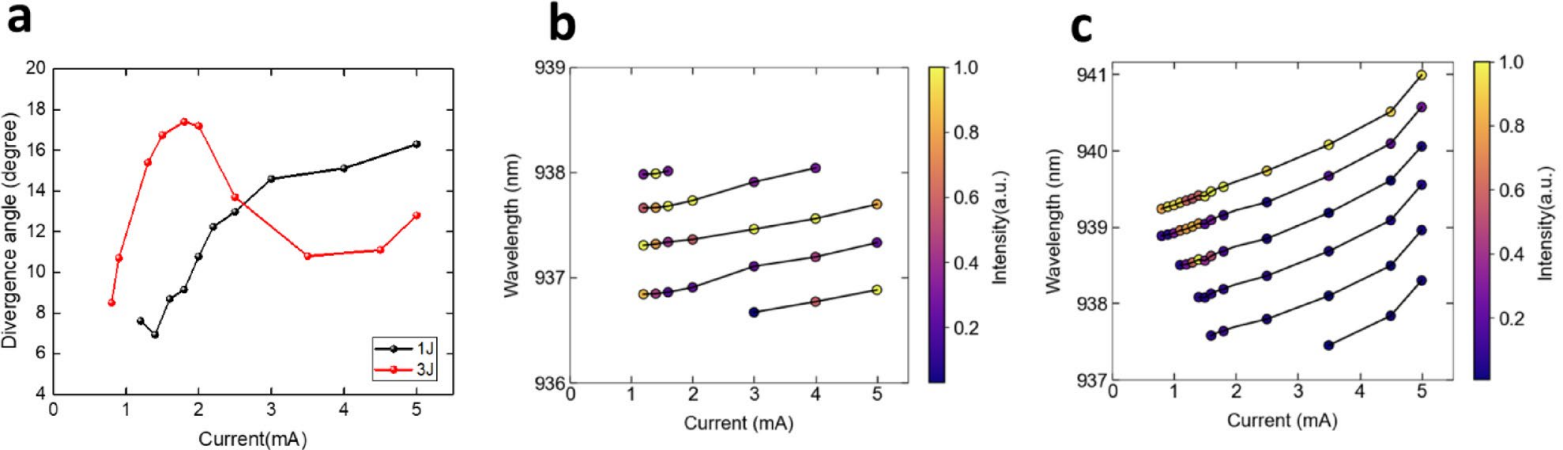
(**a**) Far-field divergence versus current for single-emitter 1 J and 3 J VCSEL (**b**) The red-shifting and relative intensity of different modes of 1 J VCSEL. (**c**) The red-shifting and relative intensity of different modes of 3 J VCSEL.

Figure 7a presents the variation in divergence angle as a function of injection current for both structures. In the 1 J structure, higher-order modes dominate the emission after the lasing threshold is reached. Furthermore, as the injection current increases, the thermal lensing effect becomes more pronounced, leading to a gradual expansion of the divergence angle, which reaches a maximum of 16.3°. In contrast, for the 3 J structure, at an injection current of 1.2 mA, the beam profile exhibits a doughnut-shaped pattern, primarily influenced by the first higher-order mode, with relatively weaker intensity at the center. As the injection current increases to 2 mA, the thermal lensing effect similarly induces an expansion in divergence angle, as observed in the 1 J structure. However, beyond 2 mA, as the injection current continues to rise, the intensities of higher-order modes progressively diminish, causing the beam profile to transition into a Gaussian shape. Consequently, the divergence angle decreases, reaching a minimum of approximately 11°. This mode transition strongly aligns with the simulated results, further substantiating the proposed mechanism. Further analysis of the device characteristics is provided and illustrates the relationship between injection current, spectral wavelength, and the relative intensity of different modes in the Fig. 7 b and c. Figure 7b corresponds to the 1 J structure, while Fig. 7c represents the 3 J structure. In the spectral data, the longest wavelength is associated with the fundamental mode, whereas shorter wavelengths correspond to higher-order modes. The results for the 1 J structure indicate that the fundamental mode exists only within a narrow current range and vanishes at higher injection currents. The emission is predominantly governed by higher-order modes, with the spectral shift favoring even higher-order modes as the current increases. For the 3 J structure, an increase in injection current leads to a gradual rise in the number of lasing modes, reaching a maximum of six modes at approximately 3.5 mA. In terms of intensity distribution, the dominant mode initially transitions from the fundamental mode to higher-order modes as the injection current increases from 0.8 mA to 1.5 mA. However, beyond 2 mA, the spectral characteristics stabilize, with the fundamental mode re-emerging as the dominant lasing mode. This result provides direct experimental evidence of mode transition, which is consistent with the observed variations in divergence angle. Notably, the divergence angle begins to decrease at this current, corresponding to a junction temperature (Tj) increase of around 4.4 °C. In the array case, a similar divergence angle reduction is observed, associated with a comparable Tj increase of approximately 4.3 °C.

## Conclusion

The characteristics of single and multi-junction VCSELs are successfully analyzed under both short pulse and CW conditions, including array and single devices. In short pulse conditions, we have found that thermal effects are successfully suppressed under nanosecond-scale operation and the peak power exceeds 120 watts in multi-junction VCSEL array. Moreover, it is shown that in both injection condition, multi-junction structures exhibit a reduction in divergence angle with increasing current, showing a stronger preference for the fundamental mode. We believe that the highly doped tunnel junction plays a critical role in this phenomenon, as the current transport path reveals a significant mitigation of current crowding near the tunnel junction. Judiciously adjusting the tunnel junction structures could be very crucial and beneficial in realizing high power VCSELs with single fundamental mode operation.

## Data Availability

The datasets used and/or analyzed during the current study available from the corresponding author on reasonable request.
